# Rapid Maxillary Expansion and Olfactory Function in Growing Subjects

**DOI:** 10.3390/children13010061

**Published:** 2025-12-31

**Authors:** Arianna Malara, Giordano Angelo Pucci, Riccardo Maurizi, Stefano Di Girolamo, Paolo Maturo, Alessia Vincenza Brescia, Raffaella Docimo, Giuseppina Laganà

**Affiliations:** 1 Department of Paediatric Dentistry (0–14 Years Old), Policlinico of Rome “Tor Vergata”, 00133 Roma, Italy; giordanoangelopucci@gmail.com (G.A.P.); alessiavincenza.brescia@ptvonline.it (A.V.B.); raffaella.docimo@ptvonline.it (R.D.);; 2 Department of Translational Medicine, Otolaryngology, University of Rome “Tor Vergata”, 00133 Roma, Italy; riccardo.maurizi@ptvonline.it (R.M.); stefano.di.girolamo@uniroma2.it (S.D.G.); 3 Department of Surgical Sciences, University of Rome “Tor Vergata”, 00133 Rome, Italy; paolo.maturo@uniroma2.it; 4 Department of Life, Health, and Health Professions Sciences, Link Campus University, 00165 Rome, Italy; g.lagana@unilink.it

**Keywords:** RME, maxillary constriction, olfactory function, growing patients

## Abstract

**Objectives:** The aim of the current study was to evaluate changes in olfactory sensitivity with Sniffin’ Sticks^®^ (Burghart Messtechnik, Germany) in patients undergoing palatal expansion. **Methods:** The study sample consisted of 20 patients enrolled from the Department of Paediatric Dentistry (0–14 years old) at the Policlinico of Rome “Tor Vergata”, according to the following inclusion criteria: negative posterior transverse interarch discrepancy ≥ 4 mm, mixed dentition phase with first permanent molars erupted and prepubertal skeletal maturation stage (CS1-2), evaluated on a lateral radiograph through the Cervical Vertebral Maturation (CVM) method. Each patient underwent a dental examination, orthopantomography and lateral cephalometric X-rays were requested, and dental impressions were taken using digital scanner. Every patient was treated with maxillary rapid expander and underwent ear, nose, and throat (ENT) assessment before and after treatment. Moreover, questionnaires before and after treatment to obtain a subjective assessment of their olfactory perception were given to all participants. **Results:** About odor identification, the analyses revealed an increase in mean scores of 1.28; however, this change, although slight, did not reach statistical significance (Z = −1.85; *p* = 0.064). In contrast, about odor discrimination, the test results indicated a statistically significant increase in the children’s scores of 3.41 (Z = −2.87; *p* < 0.001). **Conclusions:** This study supports the hypothesis that rapid maxillary expansion (RME) can improve olfactory function by enhancing nasal airway dimensions and airflow. Further studies are required to confirm these results.

## 1. Introduction

Transverse maxillary deficiency (TMD) and related palatal constriction are common concerns in orthodontic practice [1,2], and they are frequently associated with oral breathing, nasal airway limitation, and altered craniofacial growth patterns. Orthodontic interventions such as rapid maxillary expansion (RME) and surgically assisted rapid maxillary expansion (SARME) are established methods for addressing maxillary atresia and improving dental–skeletal relationships. Beyond the occlusal domain, these treatments also exert anatomical and functional changes in the nasal and nasopharyngeal airways, which may have clinical implications for nasal respiration, airway resistance, and potentially sensory modalities such as olfaction [3,4,5].

Multiple studies documented that palatal expansion increases nasal cavity width, cross-sectional area, and volume, and reduces nasal airway resistance [6,7]. A systematic review and meta-analysis found that adult patients undergoing maxillary expansion exhibited a statistically significant reduction in nasal resistance and improved subjective nasal breathing scores [8]. In children with mouth-breathing habits, an eight-year follow-up study of RME demonstrated stable improvement in nasal patency and nasomaxillary dimensions [9]. These findings support the concept that palatal expansion not only influences the occlusion but also optimizes nasal function.

Olfactory sensitivity is critically dependent on adequate airflow to the olfactory epithelium and the patency of the nasal passages. Nasal obstruction, adenoid hypertrophy, or impaired nasal ventilation have been shown to raise olfactory thresholds and reduce discrimination capacity; for instance, children with adenoid hypertrophy exhibited higher olfactory detection thresholds, which improved post-adenectomy [10]. Therefore, it is plausible that interventions that improve nasal airway geometry and airflow dynamics may enhance olfactory function.

Despite this theoretical link, the effect of palatal expansion on olfaction has been relatively under-investigated. A randomized controlled study in children (aged 6–11 years) reported significant improvement in n-butanol olfactory thresholds following RME, alongside improved peak nasal inspiratory flow (PNIF), although no change was noted in anterior active rhinomanometry values [4]. In adults undergoing SARME, a recent prospective study found that odor discrimination and identification scores improved after expansion, with threshold improvements being more pronounced in the group with limited expansion (≤6 mm) compared to larger expansions (>6 mm) [6]. These preliminary findings suggest that palatal expansion may exert a measurable effect on olfactory performance.

From the orthodontic perspective, understanding the association between palatal expansion and olfactory function is not only scientifically intriguing but clinically relevant. Patients with maxillary atresia and concomitant nasal breathing impairment may benefit from an integrated treatment approach that considers both airway and sensory outcomes. However, questions remain: What is the magnitude of olfactory improvement after expansion? Are there threshold effects in terms of expansion width or nasal valve dynamics? How durable are the changes? And to what extent should olfactory sensitivity be integrated into outcome assessment in orthodontic/otolaryngologic collaboration?

Thus, the aim of the present study is to evaluate changes in olfactory sensitivity (threshold, discrimination, identification) in patients undergoing palatal expansion and to discuss the implications for interdisciplinary care in orthodontics and otolaryngology. By providing such data, the authors hope to expand the conceptual framework of palatal expansion beyond dental correction, incorporating sensory biology and airway physiology into routine orthodontic practice.

## 2. Materials and Methods

This clinical study was carried out in accordance with the principles set out by the World Medical Assembly in the 2008 Declaration of Helsinki on medical protocols and ethics, and it was approved by the Ethical Committee of the Policlinico of Rome “Tor Vergata” (protocol number: 24/25). Written consent was obtained from both parents of every subject included in this study.

To start this study, 20 patients (12 M, 8 F, mean age 8.57 ± 1.09) were recruited from the Department of Paediatric Dentistry (0–14 years old) at the Policlinico of Rome “Tor Vergata”, according to the following inclusion criteria: negative posterior transverse inter-arch discrepancy ≥ 4 mm [11], mixed dentition phase with first permanent molars erupted and prepubertal skeletal maturation stage (CS1-2), evaluated on a lateral radiograph through the Cervical Vertebral Maturation (CVM) method [12].

Patients with previous orthodontic treatments, dental anomalies, syndromes, or systemic pathologies were excluded from this study. Patients with conditions affecting the sense of smell (e.g., chronic rhinitis, nasal polyps, severe respiratory allergies), with pre-existing smell disorders or with neuromuscular or syndromic disorders affecting respiratory function were also excluded from this investigation.

**Figure 1:**
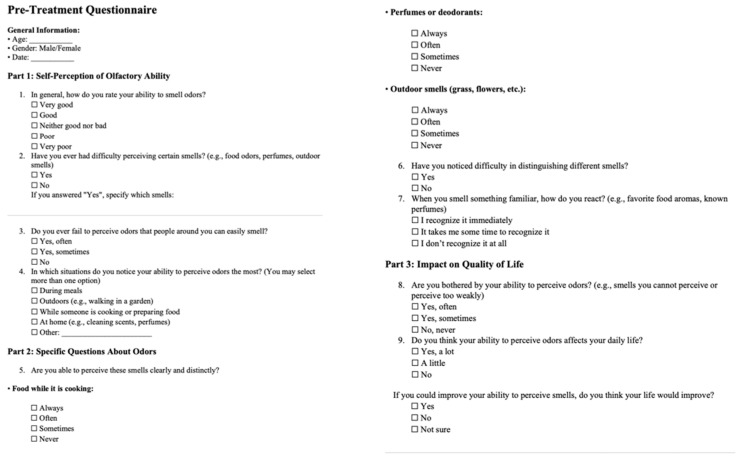
Pre-Treatment Questionnaire.

**Figure 2:**
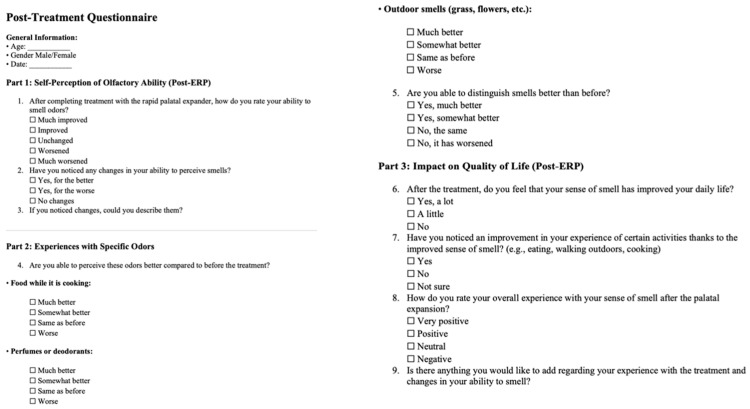
Post-Treatment Questionnaire.

Each patient underwent a dental examination, orthopantomography and lateral cephalometric X-rays were requested, and dental impressions were taken using digital scanner. Every patient was treated with maxillary rapid expander and underwent ear, nose and throat (ENT) assessment before and after treatment. Moreover, questionnaire before (Figure 1) and after (Figure 2) treatment to obtain a subjective assessment of their olfactory perception were given to all participants. These questionnaires were designed to be easy for patients to interpret and with simple answers to mark.

### 2.1. Treatment Protocol

**Figure 3:**
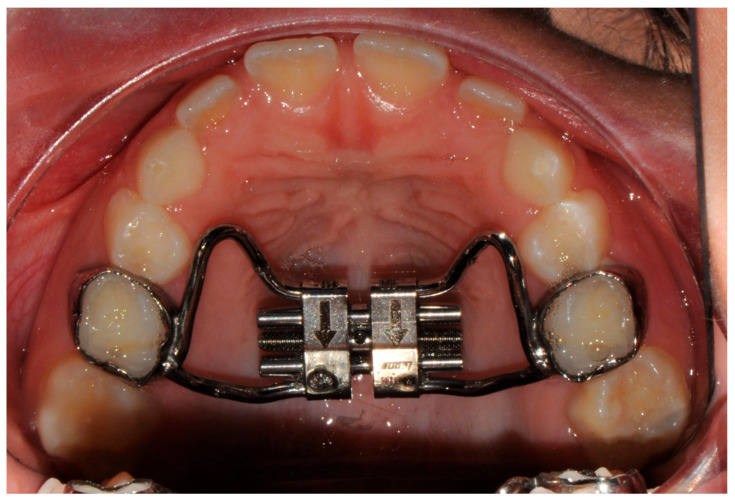
RME appliance anchored on second primary molars.

All subjects in the study group received treatment with a Hyrax Butterfly Rapid Maxillary Expander (RME made by Orthosystem, Rome, Italy) featuring a Leone® screw and bands cemented to the maxillary permanent first molars or second primary molars (Figure 3). Each patient underwent a single-phase expansion, maintaining the appliance in situ for a period of six months. Following insertion of the expander, activation was initiated and continued until overcorrection was achieved, characterized by the contact of the palatal cusps of the maxillary molars with the buccal cusps of the mandibular molars [13,14].

### 2.2. ENT Assessment

Each patient was then assessed in the ENT Department at the Policlinico of Rome “Tor Vergata”, where the first qualitative and quantitative olfactometry test was performed, a routine examination for assessing olfactory function before starting orthodontic treatment. The test was repeated once the activations were completed and again six months later.

**Figure 4:**
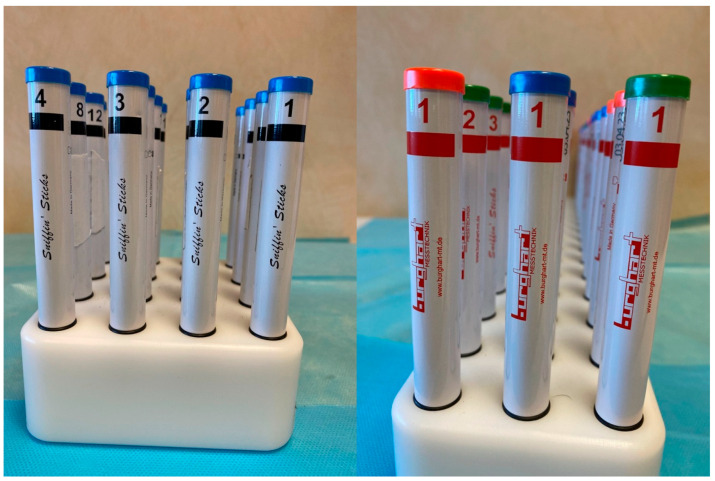
The Sniffin’ Sticks^®^ used for testing the olfactory test.

The Sniffin’ Sticks® represent a subjective olfactometric test and include a multiple forced-choice odor identification test and a threshold test [15]. The Sticks used are “Burghart Messtechnik” made in Holm, Germany (Figure 4).

In the identification test, the children are not blindfolded and they are required to smell a series of 16 pens, each impregnated with a different odorant substance, with an interval of approximately 30 s between the presentation of each pen. For each odorant, a list of four possible odor names is provided, from which the patient must choose the one that, in their judgment, most closely matches the perceived smell. The validity of the test is based on the forced-choice principle, according to which the patient must provide a response even if no odor is perceived, selecting an option at random if necessary [16]. Olfactory threshold assessment is carried out using 16 series of triplets, each consisting of three pens. Within each triplet, two pens are odorless (identified by blue and green caps), whereas the third pen (identified by a red cap) contains n-butanol at progressively increasing concentrations. Prior to testing, participants are familiarized with the odor by exposure to the lowest dilution. During the test, they are asked to identify the pen containing the odor within each triplet. Triplet 16 corresponds to the highest dilution (lowest concentration), while triplet 1 represents the lowest dilution (highest concentration) [17]. After the initial concentration level is identified, the participant is presented with the triplet at the next lower concentration. The concentration is then progressively increased until the participant fails to correctly identify the odor-containing pen in two consecutive trials. This point is recorded, after which the testing sequence is reversed and continues toward higher odor concentrations (i.e., triplets with lower numbers) until the participant again correctly identifies the odor in two consecutive trials. This staircase procedure is repeated seven times, and the olfactory threshold score is calculated as the mean of the last four reversal points [18].

### 2.3. Sample Size 

A sample size for this trial was calculated according to the method proposed by Whitehead et al. [19] For a standardized effect size of 1 (a clinically relevant change of 0.75 mm with a combined SD of 0.68 mm derived from Mavrogiannis et al. [20] for the primary out-come variable PPD at 3 months, a sample size of 17 subjects per group was required for a type I error rate of 5 and a power of 80%. To account for potential dropouts, 20 subjects per group were recruited.

### 2.4. Statistical Analysis

Data analysis was performed using SPSS statistical software version 25.0. The Wilcoxon test was conducted to verify whether the Rapid Palatal Expander was effective in influencing the components of the olfactory function (identification and discrimination).

The use of a non-parametric test was necessary due to the small sample size. Results will be considered significant if they have a *p*-value < 0.05.

Moreover, to further investigate the set of results obtained, this chapter also reports the results of the analysis of the frequency of responses to the questions included in the qualitative questionnaire designed specifically for this study.

## 3. Results

A total of 20 subjects took part in the study, 12 males (60%) and 8 females (40%) aged between 8 and 11 years (mean age = 8.57 ± 1.09) (Table 1). Wilcoxon test was performed for odor identification and odor discrimination after the RME activation and after six months expecting the same results (Table 2).

About the odor identification, the analyses revealed an increase in mean scores of 1.28; however, this change, although slight, did not reach statistical significance (Z = −1.85; *p* = 0.064). In contrast, for odor discrimination, the test results indicated a statistically significant increase in the children’s scores of 3.41 (Z = −2.87; *p* < 0.001).

The results of the children’s main responses to the questionnaire administered prior to the placement of the orthodontic appliance are reported first (Table 3). Of the participants, 60% stated that they had a good ability to perceive odors, while 40% considered their olfactory ability essentially neutral, selecting the response option “neither good nor bad.” Among the 20 participants, 65% reported experiencing difficulties in perceiving odors. 

Table 4 shows the results of the post-expansion questionnaires.

## 4. Discussion

The present study aimed to explore the correlation between transverse maxillary expansion via Rapid Maxillary Expansion (RME) and improvements in olfactory function. Findings suggest that RME may be associated with measurable enhancements in olfaction, likely mediated by changes in nasal airway dimensions and airflow dynamics. These observations align with, and extend, existing research on the airway and nasal functional changes following maxillary expansion [7].

Rapid maxillary expansion (RME) has been associated with improvements in olfactory sensitivity, likely due to increased nasal airway dimensions and reduced resistance following maxillary widening, which may enhance odorant delivery to the olfactory mucosa [7].

In the broader medical context, nasal and upper airway morphology and patency are known to influence olfactory function, as airflow dynamics and mucosal health modulate odor perception in conditions such as chronic rhinosinusitis and allergic rhinitis [21]. Individual factors including pre-existing airway obstruction, mucosal inflammation, age, and anatomical variability can therefore affect baseline olfactory performance and the extent of improvement after RME [22].

Several studies have demonstrated that rapid maxillary expansion (RME) produces measurable structural and functional changes in the nasal cavity. Increases in nasal cavity volume and minimal cross-sectional area after RME have been consistently reported in CBCT-based studies and systematic reviews [23]. Additionally, a recent computational fluid dynamics analysis conducted in children with nasal septal deviation showed that RME reduces inspiratory wall shear stress and promotes a more symmetric bilateral airflow pattern within the nasal cavities [24].

These anatomical and functional changes likely facilitate better transport of odorant molecules to the olfactory epithelium, reduce stagnation zones, and improve nasal ventilatory dynamics all of which could enhance both odor threshold (the minimum detectable concentration) and odor identification/discrimination performance.

Indeed, the data of the present study showed a statistically significant improvement in olfactory threshold and identification tasks post-expansion, consistent with the only published study to date explicitly focusing on olfaction: in adults undergoing surgically assisted RME (SARME), significant improvements in odor discrimination and identification were reported, with more modest gains in odor threshold and a nuanced relationship to expansion magnitude [6,25]. Furthermore, a pediatric prospective controlled study found improvements in N-butanol olfactory thresholds six months after RME compared to controls [24]. These convergent findings support the hypothesis that RME can yield beneficial olfactory outcomes via improved nasal airflow.

The results of the current extend prior work in two keyways. First, while many earlier investigations [26,27] focused on nasal airway resistance, patency, or upper-airway dimensions, few have explicitly measured olfactory outcomes. By adding olfactory threshold and identification tests, the present study fills a gap in the airway-olfaction literature. Second, data of the current investigation suggest that the degree of olfactory improvement may depend on baseline nasal obstruction or maxillary constriction: subjects with higher pre-treatment nasal resistance (or narrower maxilla) appeared to derive greater olfactory benefit. This mirrors earlier findings in airway studies showing greater reductions in nasal resistance in subjects with higher pre-treatment values.

## 5. Limitations

Some limitations must be acknowledged. First, the small sample size and follow-up duration limit our ability to assess long-term durability of olfactory improvements; many airway studies emphasize that long-term follow-up is lacking. Second, although olfactory threshold and identification were measured, odor discrimination or more comprehensive olfactory batteries were not assessed, nor directly assessed airflow in the olfactory cleft or use imaging/CFD, specifically for olfaction.

Finally, although it could be concluded that the underlying mechanisms from changes in nasal airway metrics, a direct assessment of odorant transport or airflow dynamics within the olfactory region would require advanced imaging techniques or computational fluid dynamics (CFD) modeling.

## 6. Clinical Implications

For clinicians, the emerging evidence suggests that in patients with maxillary transverse deficiency and concurrent nasal obstruction (e.g., mouth breathers, children with poor nasal patency), RME may offer many benefits: orthopedic maxillary correction, functional improvement in nasal breathing and even olfactory improvement. However, practitioners should manage expectations: olfactory improvement is not guaranteed, individual responses vary, and optimal results may depend on nasal anatomy and additional otolaryngologic factors. Additionally, the timing of treatment (growth stage) and careful planning of expansion magnitude appear relevant. Given the potential link between olfaction and quality of life, discussing this potential benefit with patients (and parents) may be appropriate, but with balanced emphasis on the variable nature of outcomes and need for multidisciplinary assessment (orthodontics + ENT).

In summary, the present study supports the hypothesis that Rapid Maxillary Expander can improve olfactory function, likely via improvements in nasal airflow and airway geometry. While promising, the evidence remains preliminary and further rigorous research is required to clarify the mechanisms, magnitudes of effect, and long-term stability of olfactory benefits.

## 7. Conclusions

Within the limitations of this study, rapid maxillary expansion (RME) was associated with an improvement in olfactory function in growing patients, with a statistically significant increase in odor discrimination and a non-significant positive trend in odor identification. These findings suggest that RME may favorably influence olfactory performance, likely through enhanced nasal airway dimensions and airflow.

RME should therefore be considered not only for transverse maxillary correction but also for its potential functional benefits on the upper airway. Further studies with larger samples, control groups, and longer follow-up are needed to confirm these preliminary results and clarify the clinical relevance of olfactory outcomes following palatal expansion.

## Figures and Tables

**Table 1:** Descriptive analysis of the study sample.

**Gender**	Male	12
	Female	8
	Tot.	20
**Age**	Min	8
	Max	11
	Mean	1.09

**Table 2:** Comparison between groups: results of the Wilcoxon test.

	Pre	Post	Z	*p*
**Identification**	8.29 ± 2.20	9.57 ± 1.55	−1.85	0.064
**Discrimination**	7.61 ± 0.75	11.02 ± 2.71	−2.87	<0.001

**Table 3:** Pre-expansion questionnaire results.

		Frequency	%
**In general, how do you rate your ability to smell odors?**	Very good	0	0
	Good	12	60
	Neither good nor bad	8	40
	Poor	0	0
**Have you ever had difficulty perceiving certain smells?**	Yes	13	65
	No	7	25
**If you happen to smell a familiar odor, how do you react?**	I recognize it immediately	6	30
	It takes me some time to recognize it	0	0
	I don’t recognize it at all	14	70
**When you smell something familiar, how do you react?**	Yes, often	0	0
	Yes, sometimes	6	30
	No	14	70
**Do you think your ability to perceive odors affects your daily life?**	Yes, a lot	6	30
	A little	8	40
	No	6	30
**If you could improve your ability to perceive smells, do you think your life would improve?**	Yes	0	0
	No	20	100
	Not Sure	0	0

**Table 4:** Post-expansion questionnaire results.

		Frequency	%
**After completing treatment with the rapid palatal expander, how do you rate your ability to smell odors?**	Much improved	5	25
	Improved	7	35
	Unchanged	8	40
	Worsened	0	0
	Much worsened	0	0
**Have you noticed any changes in your ability to perceive smells?**	Yes, for the better	12	60
	Yes, for the worse	0	0
	No changes	8	40
**Are you able to distinguish smells better than before?**	Yes, much better	4	20
	Yes, somewhat better	8	40
	No, the same	8	40
	No, it has worsened	0	0
**After the treatment, do you feel that your sense of smell has improved your daily life?**	Yes, a lot	3	15
	A little	9	45
	No	8	40
**Have you noticed an improvement in your experience of certain activities thanks to the improved sense of smell?**	Yes	4	20
	No	4	20
	Not sure	12	60
**How do you rate your overall experience with your sense of smell after the palatal expansion?**	Very positive	0	0
	Positive	0	0
	Neutral	20	100
	Negative	0	0

## Data Availability

The dataset is available on request from the authors.
